# A Transcriptomic View of the Proteome Variability of Newborn and Adult *Bothrops jararaca* Snake Venoms

**DOI:** 10.1371/journal.pntd.0001554

**Published:** 2012-03-13

**Authors:** André Zelanis, Débora Andrade-Silva, Marisa M. Rocha, Maria F. Furtado, Solange M. T. Serrano, Inácio L. M. Junqueira-de-Azevedo, Paulo Lee Ho

**Affiliations:** 1 Laboratório Especial de Toxinologia Aplicada (CAT/cepid), Instituto Butantan, São Paulo, Brazil; 2 Departamento de Bioquímica, Instituto de Química, Universidade de São Paulo, Brazil; 3 Laboratório de Herpetologia, Instituto Butantan, São Paulo, Brazil; 4 Centro de Biotecnologia, Instituto Butantan, São Paulo, Brazil; McGill University, Canada

## Abstract

**Background:**

Snake bite is a neglected public health problem in communities in rural areas of several countries. *Bothrops jararaca* causes many snake bites in Brazil and previous studies have demonstrated that the pharmacological activities displayed by its venom undergo a significant ontogenetic shift. Similarly, the venom proteome of *B. jararaca* exhibits a considerable variation upon neonate to adult transition, which is associated with changes in diet from ectothermic prey in early life to endothermic prey in adulthood. Moreover, it has been shown that the Brazilian commercial antibothropic antivenom, which is produced by immunization with adult venom, is less effective in neutralizing newborn venom effects. On the other hand, venom gland transcripts of newborn snakes are poorly known since all transcriptomic studies have been carried out using mRNA from adult specimens.

**Methods/Principal Findings:**

Here we analyzed venom gland cDNA libraries of newborn and adult *B. jararaca* in order to evaluate whether the variability demonstrated for its venom proteome and pharmacological activities was correlated with differences in the structure of toxin transcripts. The analysis revealed that the variability in *B. jararaca* venom gland transcriptomes is quantitative, as illustrated by the very high content of metalloproteinases in the newborn venom glands. Moreover, the variability is also characterized by the structural diversity of SVMP precursors found in newborn and adult transcriptomes. In the adult transcriptome, however, the content of metalloproteinase precursors considerably diminishes and the number of transcripts of serine proteinases, C-type lectins and bradykinin-potentiating peptides increase. Moreover, the comparison of the content of ESTs encoding toxins in adult male and female venom glands showed some gender-related differences.

**Conclusions/Significance:**

We demonstrate a substantial shift in toxin transcripts upon snake development and a marked decrease in the metalloproteinase P-III/P-I class ratio which are correlated with changes in the venom proteome complexity and pharmacological activities.

## Author Summary

*Bothrops jararaca* is one of the most abundant venomous snake species in Brazil. It is primarily a nocturnal and generalist animal, however, it exhibits a notable ontogenetic shift in diet, feeding mainly on arthropods, lizards, and amphibians (ectothermic prey) through its juvenile phase and on small mammals (endothermic animals) during adult life. Due to its broad geographical distribution, this species is responsible for the majority of the accidents by *Bothrops* genus in Brazil. Studies on envenomation cases with newborn and adult *B. jararaca* snakes have shown distinct patterns, mainly related to blood coagulation disorders, which seems to be prominent in accidents with newborn specimens. Moreover, it has been demonstrated that the Brazilian commercial antibothropic antivenom, which is produced by immunization with adult venom, is less effective in neutralizing newborn venom effects. In this study we analyzed the venom gland transcriptome of newborn snake specimens and compared the content of toxin transcripts with that of adult specimens. We demonstrate that upon *B. jararaca* development, its repertoire of mRNAs encoding toxins changes both qualitatively and quantitatively and these alterations are associated with the venom proteome profiles and pharmacological activities displayed by newborn and adult specimens.

## Introduction

In the last decade high throughput methodologies have been increasingly employed in toxinology, mainly in snake venom studies, and the results obtained by omics analysis have allowed a comprehensive view of the complexity of venom transcriptomes/proteomes [1]–[3]. Since the first publication of a snake venom gland transcriptome [4], a number of snake species had their venom gland transcriptomes revealed. These results had a tremendous contribution in snake venom protein identification by mass spectrometric analysis since no snake genome is available so far. However, all venom gland transcriptomic studies reported, including those using new generation deep sequencing technologies [5], [6], have focused on the diversity of transcripts from adult specimens. Hence, we decided to explore the venom gland transcriptome of a medically important South American snake species [7], *Bothrops jararaca*, at two different stages of its ontogeny. Previous studies have demonstrated that the ontogenetic shift in diet, from ectothermic prey in early life to endothermic prey in adulthood, and in animal developmental stages (newborn and adult) are associated with changes in the venom proteome of this species [8]–[11].

Some snake venom toxin classes undergo remarkable post-translational modifications, including proteolytic processing, oligomerization and glycosylation, which contribute to the complexity of the venom proteomes [12]–[14]. Ontogenetic variability has also been extensively reported for snake venoms [9], [11], [15]–[18] and the modification of prey types has been considered an important factor for the ontogenetic variability of venom composition [9], [11], [16], [17], [19]. Taking into consideration that snake bite was recently recognized by World Health Organization as a neglected tropical disease, the understanding of snake venom composition have remarkable implications on the improvement of anti-venom therapy and the management of snake bite [20]. Therefore, the analysis of geographical, seasonal and ontogenetic intraspecies venom variability must be taken into account in order to prepare representative venom pools for antivenom production [21].

In order to evaluate whether the variability demonstrated for the *B. jararaca* venom proteome upon newborn to adult transition was correlated or not with variations in the structure of toxin transcripts we carried out a transcriptomic analysis of venom gland from newborn and adult male and female specimens. To the best of our knowledge this is the first report on age- and gender-related variability of a snake venom gland transcriptome and the results reported here allowed insights into the ontogenetic and sexual variability of snake venom proteomes as well as provided additional information for the development of more appropriate anti-bothropic antivenom for clinical intervention.

## Methods

### Venom glands

*B. jararaca* specimens were obtained from the Herpetology Laboratory, Instituto Butantan, São Paulo, Brazil. Thirty-two animals were used, among which 20 newborns (two weeks old; 10 males and 10 females) and 12 adults (older than 3 years; 6 males and 6 females), from São Paulo State, Brazil.

Even though a few specimens are suitable to provide enough material (mRNA) for cDNA library construction, this protocol was used because the low amount of mRNA obtained for newborn specimens (data not shown).

The venom was milked and 4 days later the animals were subjected to CO_2_ anesthetization and were sacrificed by decapitation. The venom glands were carefully dissected, frozen in liquid nitrogen and kept at −80°C until use.

All animal work has been conducted in agreement with the Ethical Principles in Animal Research, adopted by the Brazilian College of Animal Experimentation and was approved by the Ethical Committee for Animal Research of Butantan Institute (protocol n° 377/07).

### cDNA libraries construction and EST generation

The integrity of total RNA was checked by discerning the 28S and 18S bands of ribosomal RNA in a formaldehyde denaturing 1% agarose gel [22]. Messenger RNA (mRNA) purification was performed on a column of oligo-dT cellulose (GE Healthcare) and the cDNAs were synthesized from 5 µg of mRNA using the Superscript Plasmid System for cDNA synthesis and Cloning (Invitrogen), and selected by size (350–600 pb and ≥600 pb) in agarose gel electrophoresis. The adapter-linked cDNAs were directionally cloned in pSPORT-1 plasmid (Invitrogen) and transformed in *Escherichia coli* DH5α. Plasmid DNA was isolated using alkaline lysis from randomly chosen clones as described [23]. DNA was sequenced on an ABI 3100 sequencer using BigDye2 kit (Applied Biosystems) with standard primers.

### Cluster assemble and identification

A homemade pipeline of EST analysis software was developed and used to remove poor quality sequences, vector, adaptors and short ESTs (<150 bp), as described elsewhere [23]. All ESTs (from adult and newborn libraries) were then assembled into clusters of contiguous sequences using the CAP3 program [24], set to join only sequences with at least 98% of base identity. The counting of ESTs originated from adult or newborn libraries in each cluster represents its level of expression in one or another group. The clusters were filtered by BLASTN against a dataset of ribosomal RNAs, mitochondrial, *E. coli* and vector sequences to mask them from statistical analysis. Each cluster was then searched against GenBank databases using BLASTX and BLASTN algorithms using Blast2Go software [25] to identify similar products with an e-value cutoff <10^−5^.Unidentified sequences and those with unpredicted function were checked for the presence of signal peptide and for orthologous occurrence in other ESTs (dbEST –www.ncbi.nml.nih.gov/nucest). A final annotation table in Microsoft Excel format was generated containing all relevant information about clusters. Methodologies of other analyses are described within the figure legends. Both single-pass reads (ESTs) and contigs are available upon request by e-mail at ijuncaze@butantan.gov.br.

## Results

### Composition of cDNA libraries

We have generated three cDNA libraries using mRNA isolated from newborn (male and female), adult male and adult female *B. jararaca* venom glands. A total of 2077 random clones including 998 from the newborn library, 559 clones from the adult male library and 520 clones from the adult female library were sequenced (Table S1). All ESTs were clustered regardless of the source of cDNA (newborn or adult). After assembling, 924 clusters were formed with lengths ranging from 153 to 2340 bp.

Slight differences in terms of abundance of toxin classes, especially Snake Venom Metalloproteinases (SVMPs) and Snake Venom Serine Proteinases (SVSPs), were noticed in comparison to a previously reported *B. jararaca* venom gland transcriptome [26]. These differences may be due to several factors such as individual and/or geographical variability. Moreover, when compared with other *Bothrops* species, the most common toxin groups (SVMPs, Bradykinin Potentiating Peptides - BPPs, SVSPs, Phospholipase A_2__–_ PLA_2_ and C-type lectins- CTLs) showed a similar pattern [4], [27], [28].

**Figure 1:**
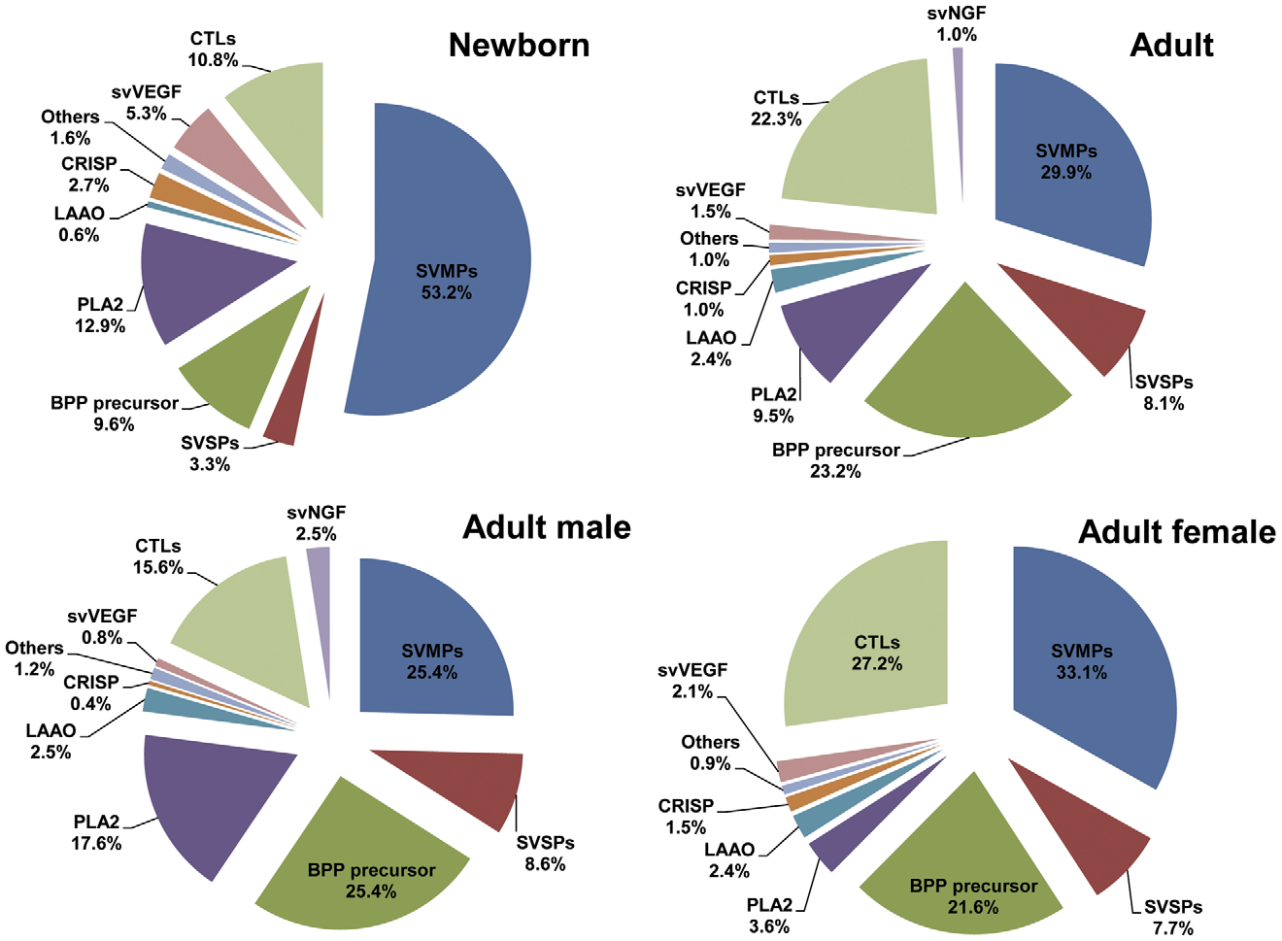
Composition of toxin transcripts in newborn and adult cDNA libraries from *B. jararaca* venom glands.

SVMPs were the most abundant toxin transcripts and showed considerable content differences between newborn (53.2%) and adult (29.9%) cDNA libraries (Figure 1). Interestingly, the amount of transcripts for snake venom Vascular Endothelium Growth Factors (svVEGF) and Cysteine-rich Secretory Proteins (CRISPs) was about 3-fold higher in the newborn venom glands (Figure 1). On the other hand, the percentage of transcripts encoding CTLs, BPP precursors, L-amino acid oxidases (LAAO) and SVSPs doubled in adult venom glands (male and female) compared to newborn ones. In addition, a few singletons corresponding to hyaluronidases, nucleotidases, phosphodiesterases and ohanin-like transcripts were also identified (Table S2).

### Sexual dimorphism in *B. jararaca* venom gland transcriptomes

The comparison of content of ESTs encoding toxins in adult male and female venom glands showed some gender-related differences. Transcripts for svVEGF, CRISP and CTL were more abundant (twice as much) in female venom glands while the number of transcripts for PLA_2_ was about five times higher in the male venom glands (Figure 1). SVMP transcripts were somewhat more abundant in the female venom gland, however, no significant difference was detected among the P-classes encoded by the clusters from the male and female libraries. Interestingly, snake venom Nerve Growth Factor (svNGF) transcripts were detected only in the male venom gland cDNA library (2.5% of total transcripts).

### Analysis of SVMP clusters

**Figure 2:**
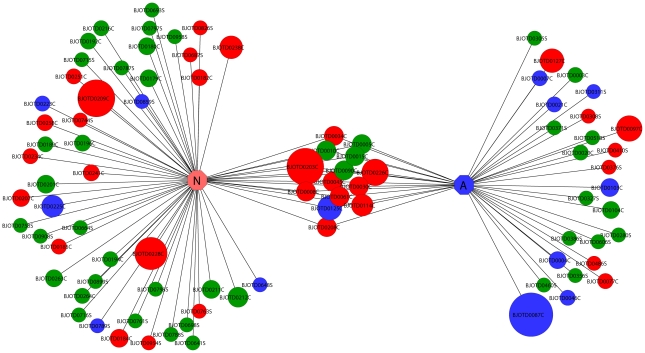
Graphical overview of SVMP clusters from newborn and adult venom gland cDNA libraries. Newborn (N) exclusive clusters are shown on the left, shared clusters in the middle and adult (A) exclusive clusters on the right. P-III class SVMPs are shown in red; P-II class SVMPs are shown in blue and SVMP clusters whose sequence matched only to UTR regions, signal peptide, pro-domain and/or catalytic domain are shown in green. The area of each circle is proportional to the number of clones comprised in that cluster. Circles are labeled as either cluster (C) or singleton (S). Graphical view generated with *Cytoscape* v. 2.8.1 [39].

As expected, after *in silico* assembly several clusters had ESTs derived from both newborn and adult cDNA libraries. However, some newborn ESTs did not overlap any EST derived from the adult cDNA library and *vice-versa*; therefore these clusters were considered as ‘exclusive’. Even though this assumption is indicative of differentially expressed genes it should be considered that it is not unequivocal and might result from a failure in the clustering of similar transcripts. Figure 2 depicts 89 SVMP clusters assembled from both newborn and adult cDNA libraries. Given the significant differences between newborn and adult SVMP expression, we have selected some clusters for a detailed analysis since SVMPs have a multi-domain architecture in which the presence of distinct domains has an implication on the biological activity of the mature protein. In this context, the only way to assign a given SVMP transcript to a SVMP class is to analyze its translated sequence inspecting for the presence of the ancillary domains (disintegrin or disintegrin-like and cysteine-rich).

**Figure 3:**
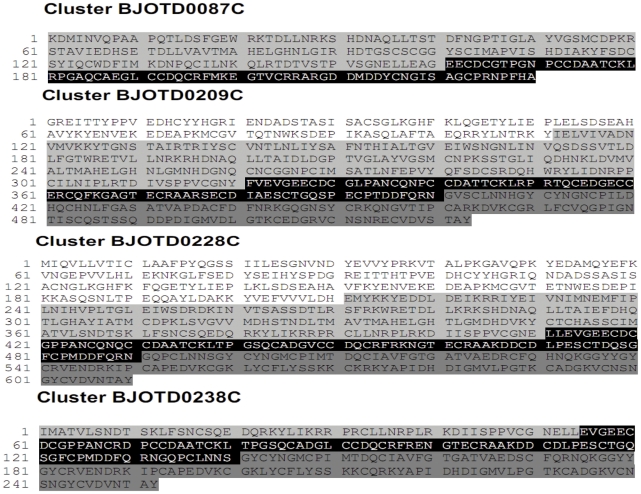
Exclusive SVMP clusters from newborn and adult venom gland cDNA libraries. Amino acid sequences deduced from the most abundant SVMP clusters (for details on cluster identification see the text). Pro (white), metalloproteinase (gray), disintegrin/disintegrin-like (black), and cysteine-rich (dark gray) domains are shaded.

The analysis of 48 newborn exclusive SVMP clusters, composed by 156 ESTs, revealed 16 clusters assigned to P-III class SVMP (84 ESTs) while the other two thirds of clusters encode P-I or P-II SVMPs (72 ESTs) (Figure 2). Among these, three clusters were significantly more expressed: BJOTD0209C (length 2.0 kb; 25 ESTs), BJOTD0228C (2.3 kb; 20 ESTs) and BJOTD0238C (1.1 kb; 10 ESTs). The translated sequences of these three clusters showed structural elements which allowed their allocation into the P-III class of SVMPs (Figure 3).

On the other hand, the analysis of adult SVMP exclusive clusters, composed by 95 ESTs, revealed that P-I and P-II class transcripts (66 ESTs) are clearly more abundant than P-III ones (30 ESTs) (Figure 2). The cluster BJOTD0087C (length 1.1 kb) was the most expressed (32 ESTs) and its corresponding translated sequence resulted in a protein with 95% similarity with the P-II class SVMP precursor of bothrostatin (gi|82219563|) from *B. jararaca*, which shows the canonical R-G-D motif typical of true disintegrins (Figure 3) [29].

### Analysis of SVSP, BPP, CTL and PLA_2_ clusters

**Figure 4:**
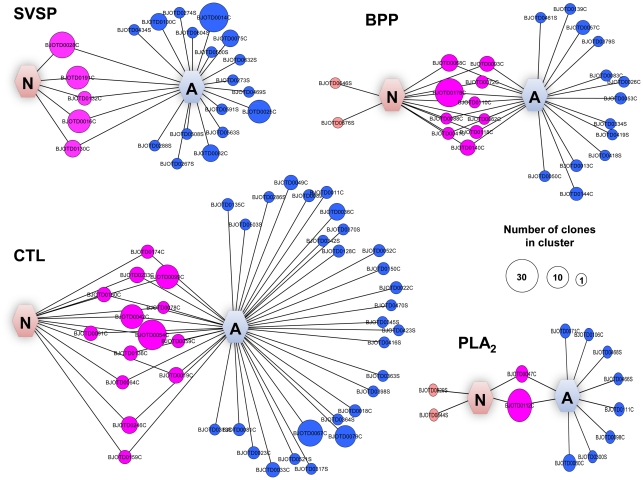
Graphical overview of toxin clusters (SVSP, CTL, PLA2 and BPP) from venom gland cDNA libraries. Newborn (N) exclusive clusters are shown on the left, shared clusters in the middle and adult (A) exclusive clusters on the right. The area of each circle is proportional to the number of clones comprised in that cluster. Circles are labeled as either cluster (C) or singleton (S). Graphical view generated with *Cytoscape* v. 2.8.1 [39].

The sequence analysis of SVSP, BPP precursors, CTL and PLA_2_ clusters revealed a lower level of complexity among these toxin classes in comparison to the SVMP clusters. Interestingly, no cluster for SVSP, BPP, CTL and PLA_2_ was found as exclusive in the newborn venom glands (Figure 4). However, a number of clusters of SVSP, BPP precursors, CTL and PLA_2_ were found as common for both newborn and adult transcriptomes whereas many clusters were identified as specific for the adult venom gland transcriptome. These data suggest that a process of diversification of transcription of these toxins classes occur upon newborn to adult transition leading to a higher degree of complexity of SVSP, BPPs, CTLs and PLA_2_ in the adult venom.

## Discussion

The analysis of mRNA transcripts by sequencing ESTs from snake venom glands cDNA libraries provides a good snapshot of the toxin arsenal of a given species. In the case of *B. jararaca*, the results on the transcriptomic analysis are broadly consistent with our previous analyses indicating a shift in SVMPs from a P-III rich newborn venom to a P-I rich adult venom [9].

The presence of transcripts for svNGF only in the male cDNA library is in accordance to the results reported by Thoenen and Barde [30] who showed that the modulation of Nerve Growth Factor (NGF) expression by male hormone testosterone was the main factor responsible for the 10-fold higher levels of this growth factor in male mouse salivary glands. Gender-related variability was already reported in *B. jararaca* venom composition and activities [31], [32]. Coagulant and hemorrhagic activities (due to the action of SVMPs or SVSPs) were detected as significantly higher in female venoms while phospholipase A_2_ and myotoxic activities were higher in male venoms [32] which is in accordance with the toxin profile observed in Figure 1. Significant differences in the BPPs content were also reported for male and female *B. jararaca* venoms. Four new BPPs were detected only in female venoms and identified by de novo sequencing as cleaved BPPs lacking the C-terminal Q-I-P-P sequence [33]. The differential abundance in toxin transcripts reported here are closely associated with the biochemical and biological properties reported for adult male and female venoms. As an interesting outcome of this study, was the fact that the content of transcripts for SVMPs, which correspond to more than 50% of the total toxin transcripts in the newborn gland, diminishes dramatically in the adult venom gland while transcripts for other important toxins classes (SVSP, CTL and BPP precursor) increase, which suggests that the snake has at its disposal a more complex toxin arsenal to deal with different types of prey in the animal adult life.

Structural diversity among SVMPs is a well known feature and is a result of the existence of distinct precursors as well as differential post-translational processing events in this toxin class [12]. The abundance of P-III class SVMP transcripts in *B. jararaca* newborn venom gland might explain the intense pro-coagulant activity that was recently reported for the newborn *B. jararaca* venom [11]. On the other hand, it was also recently shown a lower metalloproteinase activity in neonate *B. jararaca* venoms [34]. This apparent discrepancy may be explained by the differences in structural diversity of SVMPs precursors found in neonate and adult venoms. Therefore, our results suggest a diversification of the biological roles displayed by the newborn SVMPs, mainly those belonging to PIII-class, upon neonate to adult transition. The variability in *B. jararaca* venom gland transcriptome is quantitative, as illustrated by the higher content of SVMPs in the newborn venom glands. Moreover, the variability is also characterized by the structural diversity of SVMP precursors found in newborn and adult transcriptomes. The main biological implications derived from these observations were verified in the analysis of the ontogenetic variation in *B. jararaca* venom proteome [11]. The venom from newborn specimens is almost 10 times more coagulant upon human plasma than the adult venom. Furthermore, the newborn venom is able to activate Factor II and Factor X of the coagulation cascade at a much faster rate than the adult venom [11]. The coagulant activity of newborn venom is strictly related to SVMPs activity whereas in the adult venoms both SVSPs and SVMPs are involved in the generation of fibrin clots [11]. On the other hand, the hemorrhagic activity, a known feature related to P-III class SVMPs, did not vary significantly between newborn and adult venoms. According to our previous investigations [11], both newborn and adult specimens have P-III class SVMPs in their venoms, though their biological targets differ significantly. Unspecific proteolysis, a common feature displayed by P-I class SVMPs [12], [35], [36], was higher in adult venoms [11]. In fact, P-I class SVMP transcripts are highly expressed in the adult venom gland (Figure 2). In this context, the molecular basis for the explanation of distinct SVMP substrate specificities in both venoms could be related to the structural diversity verified among SVMP precursors in newborn and adult venom glands. As the snake matures and grows, the P-III/P-I class SVMP ratio decreases, while no alteration in the venom hemorrhagic activity is detected [11]. Therefore, even among newborn and adult P-III class SVMPs there are differences regarding substrate specificity; most of the newborn P-III class SVMPs tend to display pro-coagulant activity, acting upon FII/FX or both. On the other hand, among hemorrhagic P-III class enzymes there is apparently no clear difference in potency between newborn and adult venoms. Similarly, distinct profiles/contents of SVMPs were detected by the analysis of other venoms indicating a shift from a P-III rich newborn venom to a P-I rich adult venom [18], [37], [38]. Whether the P-III/P-I SVMP ratio represents an ‘ontogenetic molecular marker’ for the venoms from *Bothrops* genus remains to be evaluated in other species.

The fact that no SVSP, CTL, BPP precursor and PLA_2_ cluster exclusive for the newborn transcriptome was detected in our analysis suggests that these toxins may have less significant roles in the pharmacological activities displayed in the newborn venom. Accordingly, the higher complexity and abundance of sequences encoding SVSP, CTL, and BPP precursor in the adult venom, which occurs in parallel to the lower expression of SVMPs, indicates that these toxins are crucial for the animal to deal with the endothermic prey.

Envenomation by venomous snakes was only recently recognized by the World Health Organization as a neglected tropical disease with yearly mortality greater than several other presently recognized neglected tropical diseases [20]. Therefore, several attempts have been made in order to improve the antivenom therapy as well as the treatment of snake bite [3], [20], [21]. In this context, the knowledge of snake venom composition through high-throughput (omics) approaches provides subsidy for the understanding of the molecular targets of snake venom toxins as well as for the improvement of antivenoms. In Brazil, the human accidents caused by *Bothrops* genus are treated with anti-bothropic antivenom, which is a mixture of horse immunoglobulins obtained by immunization of horses with a pool of venom from 6 species from *Bothrops* genus (Instituto Butantan, São Paulo, Brazil). Of interesting note is the fact that no newborn venom is used in the composition of the immunogen mixture. Recently Antunes and coworkers [34] showed that the anti-bothropic antivenom was less efficient in neutralizing *in vivo* and *in vitro* activities of newborn *B. jararaca* venom. The knowledge of the transcriptome of *B. jararaca* newborn venom gland provided insights into the repertoire of toxins of this tissue at an early life stage. Furthermore we verified that there is a significant difference in terms of SVMP precursors. This feature might have an effect in the biological activities observed during the life span of *B. jararaca* species as revealed by our previous functional and proteomic analysis. In conclusion, our results were robust enough to provide the molecular basis of venom composition differences among newborn and adult *B. jararaca* as well as gender-related differences.

## Supporting Information

Table S1 **Representation of the 924 clusters assembled from 2077 newborn and adult ESTs.**
(DOC)

Table S2 **Identification of putative toxin-related clusters of** ***B. jararaca*** **venom gland ESTs.**
(XLS)
