# Supplementary material for: A Transcriptomic View of the Proteome Variability of Newborn and Adult Bothrops jararaca Snake Venoms
Source: PLoS Negl Trop Dis. 2012 Mar 13;6(3):e1554. doi: 10.1371/journal.pntd.0001554 (PMC3302817; doi:10.1371/journal.pntd.0001554)
Supplement: Table S1 — Representation of the 924 clusters assembled from 2077 newborn and adult ESTs. (DOC) [file pntd.0001554.s001.doc]

**Table S1**: Representation of the 924 clusters assembled from the 2077 sequenced ESTs from newborn and adult venom gland cDNA libraries.

| **Category** | **Number of clusters** | **Number of clones** | **Redundancy (clone/cluster)** | **Representation over matching clones** |
| --- | --- | --- | --- | --- |
| ***No hit*** | 255 | 283 | 1.1 | - |
| **Identified sequences** |  |  |  | 100% |
| Similar to toxin transcripts/proteins | 235 | 1071 | 4.5 | 59.7% |
| Similar to cellular transcripts/proteins  Unknown function | 396  38 | 677  46 | 1.7  1.2 | 37.7%  2.6% |
| **TOTAL** | 924 | 2077 |  |  |
